# High fibrinogen‐to‐albumin ratio is associated with hemorrhagic transformation in acute ischemic stroke patients

**DOI:** 10.1002/brb3.1855

**Published:** 2020-12-12

**Authors:** Yiting Ruan, Chengxiang Yuan, Yuntao Liu, Yaying Zeng, Haoran Cheng, Qianqian Cheng, Yunbin Chen, Guiqian Huang, Weilei He, Jincai He

**Affiliations:** ^1^ Department of Neurology The First Affiliated Hospital of Wenzhou Medical University Wenzhou China; ^2^ Department of Mental Health Mental Health School Wenzhou Medical University Wenzhou China

**Keywords:** acute ischemic stroke, albumin, fibrinogen, hemorrhagic transformation, inflammation

## Abstract

**Introduction:**

Hemorrhagic transformation (HT) is a complex and multifactorial complication among patients with acute ischemic stroke (AIS), and the inflammatory response has been considered as a risk factor for HT. We aimed to evaluate the stratification of FAR (fibrinogen‐to‐albumin ratio), an inflammatory biomarker, in HT patients.

**Methods:**

A total of 256 consecutive stroke patients with HT and 256 age‐ and gender‐matched stroke patients without HT were included in this study. HT during hospitalization was diagnosed by follow‐up imaging assessment and was classified into hemorrhagic infarction (HI) and parenchymal hematoma (PH) according to the recommendations of European Cooperative Acute Stroke Study II classification. Blood samples were obtained at admission.

**Results:**

Higher levels of FAR were observed in patients with HT compared with the non‐HT group [10.29 (8.39–12.95) vs. 8.60 (7.25–10.8), *p* < .001], but no significant difference was found between the PH and HI [10.88 (8.72–13.40) vs. 10.13 (8.14–12.60), *p* > .05]. Patients were assigned to groups of high FAR (≥9.51) and low FAR (<9.51) based on the optimal cut‐off value. After adjustment for potential confounders, the high FAR remained independently associated with the increased risk of HT (OR = 5.027, 95% CI = 5.027 (2.309–10.942), *p* < .001).

**Conclusions:**

High FAR was independently associated with the increased risk of HT after AIS.

## Graphical abstract

This study is the first to explore the association between fibrinogen‐to‐albumin ratio (FAR) and hemorrhagic transformation (HT) in acute ischemic stroke patients. We observed that FAR was associated with HT. The calculation of FAR may assist clinicians identify patients at elevated risk of HT and make better‐informed individualized treatment.

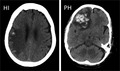

## 1 INTRODUCTION

Hemorrhagic transformation (HT) is a complex and multifactorial complication in the natural evolution of acute ischemic stroke (AIS), and often results in poor clinical outcomes, including early mortality and disability (Álvarez‐Sabín et al., 2013; Paciaroni et al., 2008). This consequence may partly relate to the aggravation of brain edema and the toxic effect of blood caused by HT (Motto et al., 1999). Therefore, early recognition and preliminary judgment of HT with novel risk factors are essential to distinguish at‐risk patients and to develop new therapies for this complication.

Inflammation has been considered as an important cause of the blood–brain barrier (BBB) disruption, which may directly lead to HT (Sandoval & Witt, 2008). Fibrinogen‐to‐albumin ratio (FAR), a new inflammatory marker, has been proposed and reported currently (Acharya et al., 2020). The predictive and prognostic value of FAR has recently been proposed in a range of diseases, including cardiovascular disease (Liang et al., 2018; Xiao et al., 2019), stroke (Acharya et al., 2020), and obstructive sleep apnea (Hizli et al., 2020). Fibrinogen (FIB) is not only an essential component of the coagulation cascade but also an acute‐phase reactant reflecting a state of systemic inflammation (Davalos & Akassoglou, 2012; Ryu et al., 2009). There were numerous studies assessed that high levels of FIB could increase the risk of stroke and consequently induce a poorer outcome (Pikija et al., 2016; Zoppo et al., 2009), and contribute to early neurologic deterioration in primary intracerebral hemorrhage (Leira et al., 2004). Conversely, albumin (ALB) has neuroprotective efficacy attribute to its antioxidant, antiapoptotic, and anti‐inflammatory properties. Studies have shown that decreased concentration of ALB contributes significantly to poor outcome in AIS patients (Cho et al., 2008).

Therefore, we hypothesized that FAR, based on a combined analysis of FIB and ALB, might serve as a predictor of HT prognosis. In the present study, we aimed to investigate the relationship between the level of FAR and HT among AIS patients.

## 2 MATERIAL AND METHODS

### 2.1 Study population

Collected from the First Affiliated Hospital of Wenzhou Medical University clinical database, all patients objectively diagnosed with HT were sampled consecutively from records between October 2011 and September 2018 in this retrospective study. Meanwhile, each HT patient was matched with a control subject (AIS patients but without HT) by age (±5 years) and gender. The exclusion criteria were as follows: (a) diagnosed with hemorrhagic stroke or transient ischemic attack; (b) history of previous stroke; (c) acute infection within 2 weeks before admission or chronic infection; (d) cancer, severe hepatic disease (alanine transaminase or aspartate transaminase >5 times the upper limit of normal) and renal diseases [estimated glomerular filtration rate < 30 ml/min) or end‐stage renal disease requiring dialysis]; (e) received intravenous thrombolysis or endovascular treatment; and (f) treated with defibrase or human albumin therapy.

The study received approval from the ethics committee of the First Affiliated Hospital of Wenzhou Medical University, and was conducted in accordance with the ethical standards of the local Research Ethics Committee on human experimentation. All procedures conformed to the Helsinki Declaration. Although could not obtain the informed consent from the patients due to the use of a retrospective study design, we were approved to collect data from our stroke registry.

### 2.2 Data collection

Patient baseline demographic characteristics were collected and documented. Clinical and laboratory information was also obtained, including atrial fibrillation, hypertension, diabetes mellitus, dyslipidemia, current cigarette smoking, and alcohol consumption, prescriptions before HT (antiplatelet, anticoagulation, and lipid‐lowering drugs), laboratory tests [leukocyte counts, platelets, hemoglobin, glucose levels (fast blood glucose), low‐density lipoprotein‐cholesterol (LDL‐C), FIB, and ALB], and blood pressure measurements were conducted within 24 hr of hospital admission. The FAR was calculated by FIB × 100%/ALB. Stroke etiology was classified according to TOAST criteria (Arsava et al., 2017). Severity of stroke was assessed by well‐trained neurologists using the National Institutes of Health Stroke Scale (NIHSS) score at admission (Kim et al., 2017). The size of each infarction area was classified as follows: less than one‐half of a lobe was defined as small, and more than one‐half of a lobe was defined as large (Álvarez‐Sabín et al., 2013).

### 2.3 Definition and classification of HT

**Figure 1:**
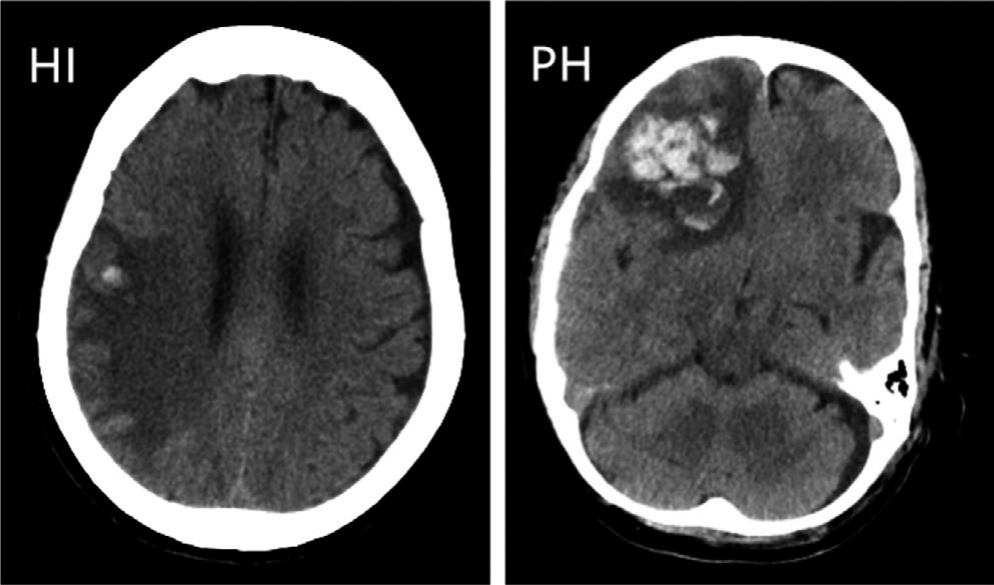
Hemorrhagic infarction and parenchymal hemorrhage. CT scan examples of hemorrhagic infarction (HI) and parenchymal hemorrhage (PH), according to the European Cooperative Acute Stroke Study II classification

All patients were examined by brain computed tomography (CT)/magnetic resonance imaging (MRI) scans at admission, at day 4 (±2), and at any neurological deterioration. HT was confirmed by follow‐up CT/MRI scans and classified as hemorrhagic infarction (HI) and parenchymal hemorrhage (PH), according to the definitions used in the European Cooperative Acute Stroke Study II classification (Hacke et al., 1998). Examples of HT subtypes were shown in Figure 1. These assessments were performed by two experienced neurologists blinded to the clinical data of patients. When there was disagreement, a third reviewer made the final decision.

### 2.4 Statistical analysis

All the eligible patients were divided into two groups: HT and non‐HT group, and the HT group was then classified as HI and PH subgroup. The receiver operating characteristic curve (ROC) was applied to determine an optimal cut‐off value for FAR score according to the Youden index. The patients were categorized into two groups according to the optimal cut‐off value of FAR for further comparisons. Continuous variables were presented as mean with standard deviation or median with interquartile range, while discrete variables were presented as counts or percentages. The continuous variables between two groups or among three or more groups were compared using *t* test, Mann–Whitney *U* test, Kruskal–Wallis test or analysis of variance when appropriate, while the categorical variables were compared using the chi‐square test. Pairwise comparisons between groups were adjusted for multiple comparisons using the Bonferroni method. Furthermore, multiple logistic regression analysis was carried out to evaluate whether FAR was associated with the incidence of HT after controlling possible confounding variables. For HT, model 1 was adjusted for age and gender; model 2 was adjusted for the variables in model 1 plus identified risk factors for HT (diabetes mellitus, systolic blood pressure (SBP), baseline NIHSS score, atrial fibrillation, current smoking, and current alcohol drinking); and model 3 was adjusted for all the variables in model 2 plus possible confounding variables (BMI, large size of the infarction area, baseline leukocyte counts, LDL‐C, the therapy of anticoagulant, antiplatelet and lipid‐lowering). Odds ratios (OR) with a 95% CI are presented. All reported *p*‐values are two‐tailed, with a *p*‐value of .05 indicating statistical significance. Statistical analysis was performed with SPSS software for Windows version 25.0 (SPSS Inc.).

## 3 RESULTS

### 3.1 Baseline characteristics of patients in HT group and Non‐HT group

A total of 256 HT patients and 256 non‐HT patients with AIS were included in the study. The baseline demographic, clinical, and laboratory characteristics of the study population are summarized in Table 1. Comparison of variables between the two groups showed that the rate of atrial fibrillation, baseline leukocyte counts, glucose levels, NIHSS scores, FAR levels, the proportion of Large size of the infarction area increased in the HT group, while BMI, SBP, platelets counts and LDL‐C levels decreased (*p* all < .05). Besides, compared with non‐HT patients, HT patients were more likely to receive anticoagulation treatment and were less likely to undergo antiplatelet or lipid‐lowering therapies.

**Table 1:** Differences of the baseline characteristics in AIS patients with and without HT

Variables	Non‐HT (*n* = 256)	HT (*n* = 256)	*p*‐Value*
FAR, median (IQR)	8.60 (7.25–10.8)	10.29 (8.39–12.95)	<.001***
Demographic parameters			
Age (years), median (IQR)	71.0 (60.5–77.0)	71.0 (62.0–78.0)	.955
Male, *n* (%), median (IQR)	178 (69.5%)	178 (69.5%)	1
BMI (kg/m^2^), median (IQR)	23.4 (21.6–25.6)	22.7 (20.8–24.5)	.017*
Marital status			
Married, *n* (%)	238 (93.0%)	247 (96.5%)	.075
Vascular risk factors			
History of atrial fibrillation, *n* (%)	26 (10.2%)	97 (37.9%)	<.001***
History of hypertension, *n* (%)	178 (69.5%)	163 (63.7%)	.160
History of diabetes, *n* (%)	75 (29.3%)	64 (25.0%)	.274
History of dyslipidemia, *n* (%)	15 (5.9%)	20 (7.8%)	.381
Current smoking, *n* (%)	78 (30.5%)	81 (31.6%)	.774
Current drinking, *n* (%)	65 (25.4%)	75 (29.3%)	.321
Biochemistry and vital signs on admission			
Large size of the infarction area, *n* (%)	6 (2.3%)	90 (35.2%)	<.001***
Baseline SBP (mmHg), median (IQR)	156.0 (144.0–170.0)	148.5 (135.0–164.0)	<.001***
Baseline DBP (mmHg), median (IQR)	81.0 (73.0–91.5)	82.0 (73.3–90.0)	.563
Leukocyte counts (×109/L), median (IQR)	6.57 (5.57–7.72)	7.93 (6.35–9.90)	<.001***
Platelets (×109/L), median (IQR)	201.0 (173.0–236.5)	183.0 (147.0–231.0)	.003**
Hemoglobin (g/L), median (IQR)	136.0 (125.0–147.8)	136.0 (126.0–146.0)	.659
Glucose levels (mmol/L), median (IQR)	5.1 (4.6–6.4)	5.75 (4.80–7.08)	<.001***
LDL‐C (mmol/L), median (IQR)	2.65 (2.09–3.34)	2.43 (1.72–3.06)	.001**
NIHSS on admission, median (IQR)	3.0 (2.0–5.0)	10.0 (6.0–13.0)	<.001***
Stroke mechanisms			
Atherosclerotic, *n* (%)	233 (91.0%)	172 (67.2%)	<.001***
Cardioembolic, *n* (%)	19 (7.4%)	83 (32.4%)	
Lacunar, *n* (%)	3 (1.2%)	1 (0.4%)	
Other causes, *n* (%)	1 (0.4%)	0 (0%)	
Initial treatment before HT			
Antiplatelets, *n* (%)	231 (91.3%)	137 (53.5%)	<.001
Anticoagulants, *n* (%)	24 (9.5%)	80 (31.3%)	<.001
Lipid‐lowering agents, *n* (%)	241 (95.6%)	219 (85.5%)	<.001

Abbreviations: BMI, body mass index; DBP, diastolic blood pressure; FAR, fibrinogen‐to‐albumin ratio; HT, hemorrhagic transformation; IQR, interquartile range; LDL‐C, low‐density lipoprotein‐cholesterol; NIHSS, National Institutes of Health Stroke Scale; SBP, systolic blood pressure.

* *p* < .05;

** *p* < .01;

*** *p* < .001

### 3.2 Baseline characteristics of all patients in the high and low FAR groups

**Figure 2:**
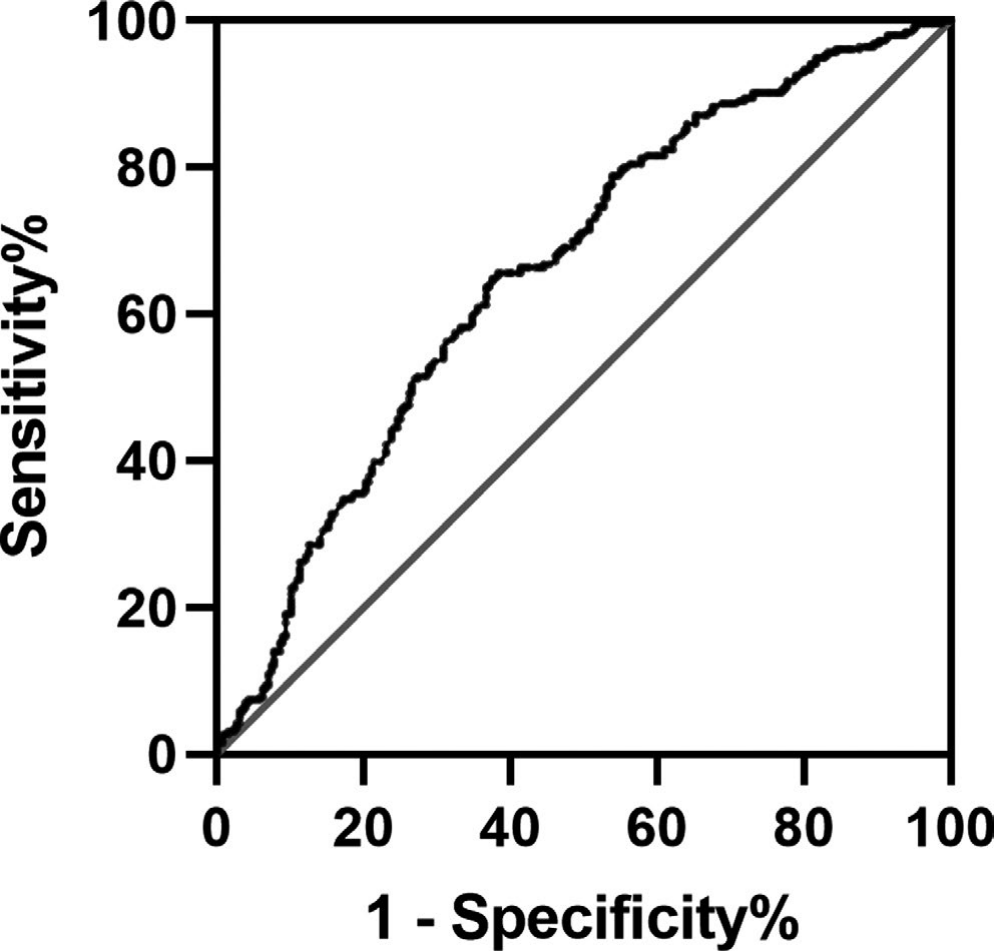
ROC analysis of fibrinogen‐to‐albumin ratio for predicting hemorrhagic transformation

The optimal cut‐off value for FAR score determined by the ROC curve was 9.51 (the area under the ROC curve: 0.661, 95% CI: 0.614–0.708, *p* < .001) with a sensitivity of 62.5% and specificity of 64.8% (Figure 2). The patients were assigned to two groups [High FAR (≥9.51) or Low FAR (<9.51)]. The characteristics of the AIS participants between the two FAR groups, including the demographic, clinical, and laboratory characteristics, are summarized in Table 2. No significant differences were observed in terms of most characteristics except for age (*p* < .001), BMI (*p* = .006), large size of the infarction area (*p* < .001), leukocyte counts (*p* < .001), hemoglobin (*p* = .003), and NIHSS scores on admission (*p* = .004).

**Table 2:** Baseline characteristics of patients with acute ischemic stroke in the high (≥9.51) and low (<9.51) FAR groups

Variables	All patients	Low FAR (*n* = 261)	High FAR (*n* = 251)	*p*‐Value*
FAR, median (IQR)	9.18 (7.61–11.3)	7.71 (6.89–8.58)	11.65 (10.65–13.59)	<.001***
Demographic parameters				
Age (years), median (IQR)	71.0 (61.0–77.0)	69.0 (58.0–75.0)	72.0 (63.0–80.0)	.001**
Male, *n* (%), median (IQR)	356 (69.5%)	186 (71.3%)	170 (67.7%)	.385
BMI (kg/m^2^), median (IQR)	23.1 (21.0–25.2)	23.5 (21.6–25.6)	22.7 (20.8–24.5)	.006**
Marital status				
Married, *n* (%)	485 (94.7%)	246 (94.3%)	239 (95.2%)	.625
Vascular risk factors				
History of atrial fibrillation, *n* (%)	123 (24.0%)	54 (20.7%)	69 (27.5%)	.072
History of hypertension, *n* (%)	341 (66.6%)	164 (62.8%)	177 (70.5%)	.065
History of diabetes, *n* (%)	139 (27.1%)	66 (25.3%)	73 (29.1%)	.334
History of dyslipidemia, *n* (%)	35 (6.8%)	19 (7.3%)	16 (6.4%)	.685
Current smoking, *n* (%)	159 (31.1%)	75 (28.7%)	84 (33.5%)	.248
Current drinking, *n* (%)	140 (27.3%)	73 (28.0%)	67 (26.7%)	.746
Biochemistry and vital signs on admission				
Large size of the infarction area, *n* (%)	96 (18.8%)	31 (11.9%)	65 (25.9%)	<.001***
Baseline SBP (mmHg), median (IQR)	154.0 (141.0–169.5)	155.0 (141.0–169.8)	150.0 (138.0–166.0)	.093
Baseline DBP (mmHg), median (IQR)	81.0 (73.0–90.0)	82.5 (75.0–92.0)	80.0 (72.0–89.0)	.099
Leukocyte counts (×109/L), median (IQR)	6.86 (5.62–8.25)	6.77 (5.60–8.30)	7.49 (6.27–9.36)	<.001***
Platelets (×109/L), median (IQR)	197.0 (168.0–235.5)	192.0 (160.2–231.8)	196.0 (162.0–291.0)	.704
Hemoglobin (g/L), median (IQR)	136.0 (125.0–147.0)	140.0 (127.2–148.0)	135.0 (123.3–155.0)	.003**
Glucose levels (mmol/L), median (IQR)	5.20 (4.60–6.50)	5.30 (4.60–6.58)	5.45 (4.70–6.88)	.374
LDL‐C (mmol/L), median (IQR)	2.65 (2.00–3.32)	2.50 (1.89–3.13)	2.63 (1.88–3.35)	.498
NIHSS on admission, median (IQR)	4.0 (2.0–8.0)	5.0 (2.0–9.0)	6.0 (3.0–11.0)	.004**
Stroke mechanisms				
Atherosclerotic, *n* (%)	405 (79.1%)	217 (83.1%)	188 (74.9%)	.056
Cardioembolic, *n* (%)	102 (19.9%)	40 (15.3%)	62 (24.7%)	
Lacunar, *n* (%)	4 (0.8%)	3 (1.1%)	1 (0.4%)	
Other causes, *n* (%)	1 (0.2%)	1 (0.4%)	0 (0%)	
Initial treatment before HT				
Antiplatelets, *n* (%)	368 (72.3%)	187 (72.2.%)	181 (72.4%)	.960
Anticoagulants, *n* (%)	104 (20.4%)	53 (20.5%)	51 (20.4%)	.986
Lipid‐lowering agents, *n* (%)	460 (90.6%)	235 (91.1%)	225 (90.0%)	.676

Abbreviations: BMI, body mass index; DBP, diastolic blood pressure; FAR, fibrinogen‐to‐albumin ratio; HT, hemorrhagic transformation; IQR, interquartile range; LDL‐C, low‐density lipoprotein‐cholesterol; NIHSS, National Institutes of Health Stroke Scale; SBP, systolic blood pressure.

* *p* < .05;

** *p* < .01;

*** *p* < .001

### 3.3 Association between the level of FAR and HT

**Figure 3:**
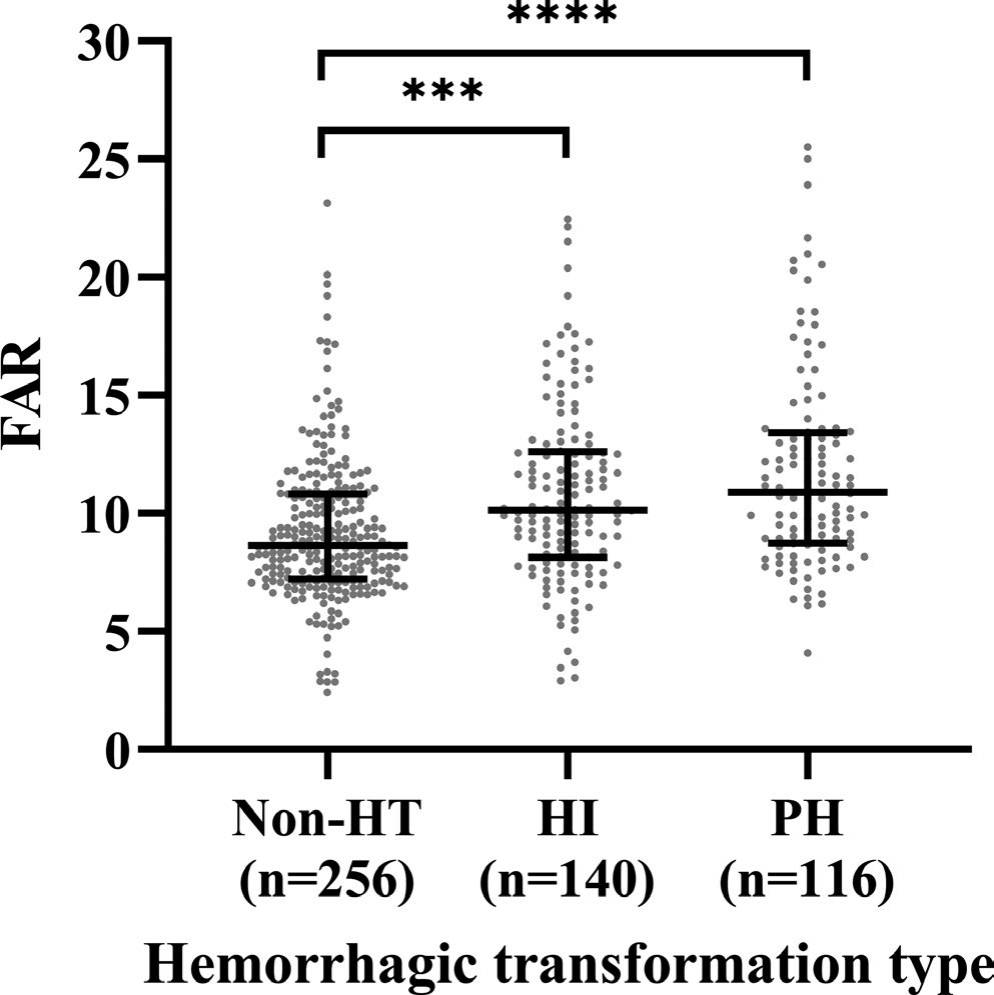
The levels of FAR in the subcategorized groups of HT. (a) The FAR was higher in patients with HI than those with non‐HT [10.13 (8.14–12.60) vs. 8.64 (7.21–10.80), *p* < .001]; (b) The FAR was higher in patients with PH than those with non‐HT [10.88 (8.72–13.40) vs. 8.64 (7.21–10.80), *p* < .001]. The *p*‐value has been adjusted using Bonferroni correction. FAR, fibrinogen‐to‐albumin ratio; HI, hemorrhagic infarct; HT, hemorrhagic transformation; PH, parenchymal hematoma; ***p < .001

**Figure 4:**
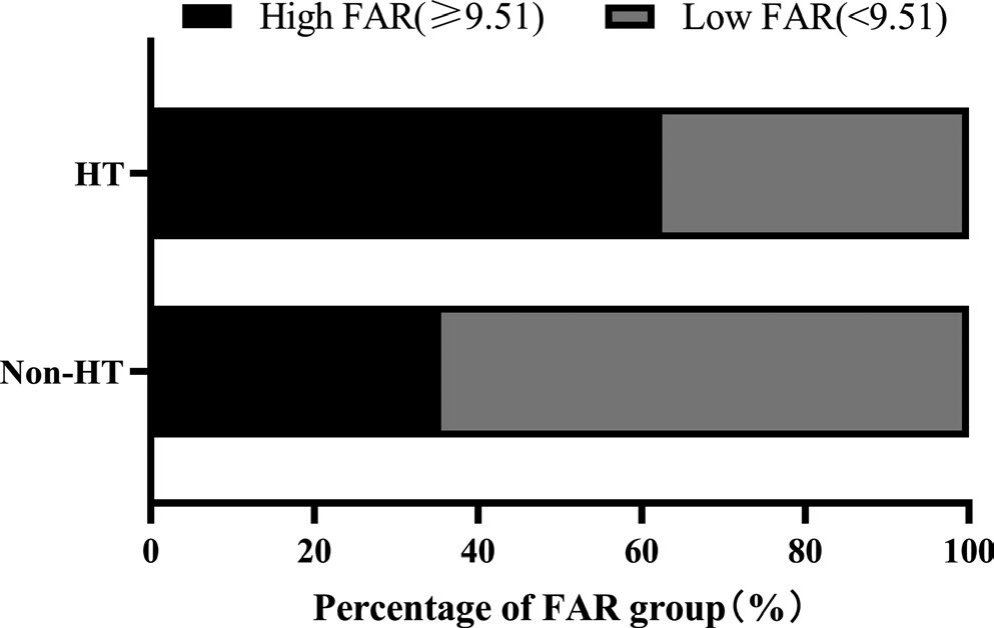
The percentage of FAR group in HT and non‐HT groups. FAR, fibrinogen‐to‐albumin ratio; HT, hemorrhagic transformation

Among the HT patients, 140 patients had HI and 116 patients had PH. Differences of FAR level among the non‐HT, HI and PH groups were statistically significant [HI vs. non‐HT: 10.13 (8.14–12.60) vs. 8.64 (7.21–10.80), *p* < .001; PH vs. non‐HT: 10.88 (8.72–13.40) vs. 8.64 (7.21–10.80), *p* < .001] (Figure 3). Indeed, there were significant differences observed between the HT and non‐HT patients according to different FAR groups. Patients with HT were more likely to have high FAR than those without HT (62.5% vs. 35.5%, *p* < .001) (Figure 4). In Table 3, binary logistic regression analysis was performed with HT occurrence as the dependent variable and the low FAR was used as the reference. After adjusting for age, gender, the high FAR (>9.51) was independently associated with HT (model 1: OR = 3.022, 95% CI = 2.109–4.331, *p* < .001). After adjusting for other potential confounders including diabetes mellitus, SBP, baseline NIHSS score, atrial fibrillation, large size of the infarction area, current smoking, and current alcohol drinking, the high FAR was still remained significant independently associated with the prevalence of HT (model 2: OR = 2.933, 95% CI = 1.784–4.823, *p* < .001; model 3: OR = 5.027, 95% CI = 2.309–10.942, *p* < .001). In logistic regression model 3, we also found that atrial fibrillation, NIHSS scores and large size of the infarction area were associated with HT (OR = 2.707, 95% CI = 1.127–6.504, *p* = .026; OR = 1.363, 95% CI = 1.243–1.494, *p* < .001; OR = 7.304, 95% CI = 2.151–24.804, *p* = .001). The antiplatelet therapy was protective in HT (OR = 0.090, 95% CI = 0.036–0.227, *p* < .001). When we repeated the binary logistic regression analyses taking FAR as continuous variables instead of categorical variables, similar results were obtained, as shown in Table S1.

**Table 3:** Binary logistic model to explore the risk factors of HT in stroke patients

	Model 1		Model 2		Model 3	
	OR (95% CI)	*p*‐Value	OR (95% CI)	*p*‐Value	OR (95% CI)	*p*‐Value
High FAR	3.022 (2.109–4.331)	<.001***	2.933 (1.784–4.823)	<.001***	5.027 (2.309–10.942)	<.001***
Atrial fibrillation			3.331 (1.794–6.186)	<.001***	2.707 (1.127–6.504)	.026*
Baseline NIHSS			1.383 (1.293–1.479)	<.001***	1.363 (1.243–1.494）	<.001***
Large size of the infarction area, *n* (%)			12.462 (4.803–32.330)	<.001***	7.304 (2.151–24.804）	.001**
Antiplatelet						<.001***

Note:

Model 1: adjusted for age, gender. Model 2: adjusted for covariates from Model 1 and further adjusted for identified risk factors for HT (diabetes mellitus, systolic blood pressure, baseline NIHSS score, atrial fibrillation, current smoking, and current alcohol drinking, large size of the infarction area). Model 3: adjusted for covariates from Model 2 and further adjusted for BMI, baseline leukocyte counts, LDL‐C, the therapy of anticoagulant, antiplatelet and lipid‐lowering. FAR, fibrinogen‐to‐albumin ratio; HT, hemorrhagic transformation; OR, odd ratio; CI, confidence interval; NIHSS, National Institutes of Health Stroke Scale; LDL‐C, low‐density lipoprotein‐cholesterol;

* *p* < .05;

** *p* < .01;

*** *p* < .001.

## 4 DISCUSSION

Based on our findings, we are the first to directly link FAR levels to HT among patients with AIS. Our results suggest that FAR level is positively correlated with HT, and high FAR was independently associated with the increased risk of HT even after adjustment for all potential confounders.

Structurally, evidence suggests that the fundamental mechanism leading to HT is the disruption of BBB (Khatri et al., 2012). Another study found that diffuse, mild BBB disruption in ischemic brain was reversible with early reperfusion, while focal, severe BBB disruption after sustained ischemia may confer an increased risk of HT. Acute cerebral ischemia activates a cascade of cellular and metabolic imbalances that may potentially cause oxidative injury and the BBB damage (Simpkins et al., 2016). Neuroinflammation following ischemic stroke has repeatedly been shown to disrupt the BBB, such as pro‐inflammatory cytokines and chemokines, oxidative, matrix metalloproteinases, and inflammatory cells (Kochetov et al., 2010). Blood‐derived substances are able to permeate the damaged BBB, leading to vasogenic edema, HT, and increased patient mortality (Lu et al., 2018).

Fibrinogen, known as an acute‐phase plasma protein, is capable of entering the central nervous system through a leaky BBB and likely contributes to neuronal injury, neuroinflammation, and immune cell recruitment in the nervous system (Merlini et al., 2019). Early studies revealed that FIB was deposited in the brain in a wide range of neurological diseases, such as multiple sclerosis (Miranda Acuña et al., 2017), Alzheimer's disease (van Oijen et al., 2005), and traumatic central nervous system injury (Jenkins et al., 2018). Some studies showed FIB depleting agents delivered in AIS patients could reduce neurological impairment and improve the quality of life in patients with AIS (Chen et al., 2018; Guo et al., 2006). Several studies showed that hyperfibrinogenemia might increase the risk of HT after intravenous thrombolysis with rt‐PA in acute cerebral infarction (Liang et al., 2012; Trouillas et al., 2004; Xu et al., 2017). However, there has only been one clinical trial showed that high FIB concentration is associated with the risk of HT in AIS patients without thrombolytic treatment (Kochetov et al., 2010). Although the underlying mechanisms are currently unclear, there are several possible explanations account for the association observed. First, binding of FIB to the receptor on microglia/macrophage stimulates the secretion of pro‐inflammatory cytokines and chemokines and the release of reactive oxygen species, which is further associated with BBB disruption and brain injury (Petersen et al., 2018). Meanwhile, increased pro‐inflammatory cytokine release after stroke, especially interleukin‐6, could trigger the high‐expression of FIB as well (Armulik et al., 2010). Second, an enhanced concentration of FIB could activate endothelial matrix metalloproteinase‐9, which is known to cause vascular remodeling and an increase in its permeability (Muradashvili et al., 2011). Finally, as a component of the perivascular extracellular matrix, FIB could increase BBB permeability via direct actions on brain endothelial cells (Lominadze et al., 2010; Petersen et al., 2018). Overall, we propose that FIB leakage upon BBB disruption exacerbated inflammatory responses and aggravated BBB impairment after ischemia, which eventually leads to HT.

Furthermore, ALB has been considered as a negative inflammatory protein with neuroprotective effect. Moreover, the protein can persist for a long time to aid in the recovery of brain ischemia due to its long half‐life and long duration of action (Belayev et al., 2001). Several studies have indicated that hypoalbuminemia was associated with the development of HT (Che et al., 2017; Wang et al., 2019) and the poor functional outcome of stroke (Rajmohan, 2020). Much researches have shown that ALB administration could effectively reduce infarction volumes and brain edema, as well as BBB permeability (Belayev et al., 2001; Park et al., 2017). Based on previous research, we speculate that ALB may exert neuroprotection by antioxidant activity, anti‐inflammatory activity, regulation of microvascular permeability, and inhibition of endothelial apoptosis. Besides, serum ALB can directly reduce BBB permeability due to its high‐molecular weight (Prajapati et al., 2011). We propose that these neuroprotective effects of serum ALB may also reduce the risk of HT in AIS patients.

In particular, interaction of ALB with FIB may dampen FIB activity and decrease the fibrin build‐up (Galanakis, 1992). Owing to such synergy, the FAR is correlated with inflammation and has been proven to be a promising predictor of disease progression and poor long‐term prognosis in patients with coronary artery disease (Çetin et al., 2019; Xiao et al., 2019), malignancies (Zhang & Xiao, 2019), and stroke (Acharya et al., 2020). Our findings suggest that calculation of FAR on admission may assist clinicians in identifying patients at elevated risk of HT. Whether alterations in therapy such as defibrase agents based on a high FAR will lead to favorable outcomes remains to be seen. However, defibrase therapy also plays a role in HT because of its coagulation effect, which needs to be weighed by the clinician.

Our findings are consistent with previous studies that have demonstrated that atrial fibrillation, baseline NHISS score, and large size of the infarction area were risk factors of HT (Álvarez‐Sabín et al., 2013; Dang et al., 2019; Paciaroni et al., 2008). In addition, our study showed that antiplatelet therapy was related to low risk of HT. One possible contributor to this phenomenon may be that patients treated with antiplatelet therapy have a lower proportion of cardiogenic embolism, which is closely related to HT.

There are some limitations in this study. First, our study is a retrospective single‐center analysis and there may be subjective selection bias, reducing the reliability of results. It is important to examine the findings in the multi‐center study. Also, matching procedures may introduce potential selection bias which was inevitable. Second, the FAR level was recorded only once at admission, which limited the observation of dynamic change overtime. Third, we only included the early outcomes during hospitalization, and the long‐term prognoses could not be obtained. More long‐term follow‐up data are required. Fourth, other causes of stroke were rare indeed in our study, we will collect more data of stroke patients of other rare causes and include them in future studies. Finally, further studies are needed to provide better insights into the inflammatory response that may potentiate risk of HT occurrence.

## 5 CONCLUSION

In conclusion, our study demonstrates that FAR is associated with HT after AIS, but the severity of HT (HI or PH) cannot be distinguished. A better understanding of the role of FAR can help clinicians have a preliminary judgment on the risk of HT and make better‐informed individualized treatment to reduce harm from this complication.

## CONFLICT OF INTEREST

The authors declare no financial or other conflicts of interest.

## AUTHOR CONTRIBUTIONS

Yiting Ruan and Jincai He designed the study. Yiting Ruan did the statistical analyses and wrote the manuscript draft. Chengxiang Yuan, Yuntao Liu prepared figures. Chengxiang Yuan, Yuntao Liu, Yaying Zeng, Haoran Cheng, Qianqian Cheng, Yunbin Chen, Guiqian Huang, Weilei He screened and extracted data. Jincai He supervised study. All authors have made an intellectual contribution to the manuscript and approved the submission.

### Peer Review

The peer review history for this article is available at https://publons.com/publon/10.1002/brb3.1855.

## Supporting information

Table S1

## Data Availability

The data sets generated in the current study are available from the corresponding author on reasonable request without undue reservation.
