# Supplementary material for: High fibrinogen‐to‐albumin ratio is associated with hemorrhagic transformation in acute ischemic stroke patients
Source: Brain Behav. 2020 Dec 12;11(1):e01855. doi: 10.1002/brb3.1855 (PMC7821560; doi:10.1002/brb3.1855)
Supplement: Supplementary file 1 — Table S1 [file BRB3-11-e01855-s001.docx]

**Supplementary Table 1** Multivariable logistic model to explore the risk factors of HT in stroke patients

|  | Model 1 | |  | Model 2 | |  | Model 3 | |
| --- | --- | --- | --- | --- | --- | --- | --- | --- |
|  | **OR (95% CI)** | ***P*-value** |  | **OR (95% CI)** | ***P*-value** |  | **OR (95% CI)** | ***P*-value** |
| FAR | 1.179（1.115-1.247） | <0.001^***^ |  | 1.160 (1.075-1.251) | <0.001^***^ |  | 1.205 (1.084-1.339) | 0.001^**^ |
| Atrial fibrillation |  |  |  | 3.195(1.722-5.926) | <0.001^***^ |  | 2.475(1.014-6.045) | 0.047^*^ |
| Baseline NIHSS |  |  |  | 1.379(1.290-1.475) | <0.001^***^ |  | 1.354(1.235-1.484) | <0.001^***^ |
| Large size of the infarction area, n (%) |  |  |  | 12.927(4.965-33.656) | <0.001^***^ |  | 7.628(2.128-27.338) | 0.002^**^ |
| Antiplatelet |  |  |  |  |  |  | 0.102(0.042-0.246) | <0.001^***^ |

NOTE: Model 1: adjusted for age, gender. Model 2: adjusted for covariates from Model 1 and further adjusted for identified risk factors for HT (diabetes mellitus, systolic blood pressure, baseline NIHSS score, atrial fibrillation, Current smoking and current alcohol drinking, Large size of the infarction area). Model 3: adjusted for covariates from Model 2 and further adjusted for BMI, baseline leukocyte counts, LDL-C, the therapy of anticoagulant, antiplatelet and lipid-lowering. FAR, fibrinogen to albumin ratio; HT, hemorrhagic transformation; OR, odd ratio; CI, confidence interval; NIHSS, National Institutes of Health Stroke Scale; LDL-C, low-density lipoprotein-cholesterol; *P <0.05; **P <0.01; ***P < 0.001
